# Flavonoids as Phytoestrogenic Components of Hops and Beer

**DOI:** 10.3390/molecules25184201

**Published:** 2020-09-14

**Authors:** Tomasz Tronina, Jarosław Popłoński, Agnieszka Bartmańska

**Affiliations:** Department of Chemistry, Wrocław University of Environmeuintal and Life Sciences, Norwida 25, 50-375 Wrocław, Poland; jaroslaw.poplonski@upwr.edu.pl (J.P.); agnieszka.bartmanska@upwr.edu.pl (A.B.)

**Keywords:** phytoestrogens, prenylflavonoids, biological activities, hop, beer

## Abstract

The value of hops (*Humulus lupulus* L.) in beer production has been undisputed for centuries. Hops is rich in humulones and lupulones which gives the characteristic aroma and bitter taste, and preserves this golden drink against growing bacteria and molds. Besides α- and β-acids, the lupulin glands of hop cones excrete prenylated flavonoids, which exhibit a broad spectrum of biological activities and therefore has therapeutic potential in humans. Recently, interest in hops was raised due to hop prenylated flavanones which show extraordinary estrogen activities. The strongest known phytoestrogen so far is 8-prenylnaringenin (8-PN), which along with 6-prenylanaringenin (6-PN), 6,8-diprenylnaringenin (6,8-DPN) and 8-geranylnaringenin (8-GN) are fundamental for the potent estrogen activity of hops. This review provides insight into the unusual hop phytoestrogens and shows numerous health benefits associated with their wide spectrum of biological activities including estrogenic, anticancer, neuropreventive, antinflamatory, and antimicrobial properties, which were intensively studied, and potential applications of these compounds such as, as an alternative to hormone replacement therapy (HRT).

## 1. Hop

Hop (*Humulus lupulus* L.) is a vine belonging to the genus *Humulus* from the family *Cannabaceae* [1]. Hops are dioecious perennials found in the northern hemisphere and reach a height of 7–8 m. Plants are cultivated in North America mainly in Idaho, Oregon, and Washington and in Europe in Germany, Great Britain, Poland, and the Czech Republic. In Asia, cultivation takes place in some areas of China, and to a limited extent in Japan, and in the southern hemisphere, in some regions of Australia and New Zealand [2].

The historic use of the female inflorescences of *Humulus lupulus* (hop) is interesting because its technical properties—as the source of bitter taste and preservation of beer—were discovered in the Middle Ages. According to Wiesner (1883), so-called herbal beers were produced in the years 1300–1500 [2].

In traditional medicine, hops have long been used to treat sleep disorders, as a stomach remedy, and as an antibacterial and antifungal agent [3]. *Humulus lupulus* attracted special attention because it contains many dietary phytochemicals that can be used for medicinal purposes, due to their antiseptic, (an)aphrodisiac, anticancer, antiplatelet, antidiuretic, antiinflammatory and sedative properties [4].

**Figure 1:**
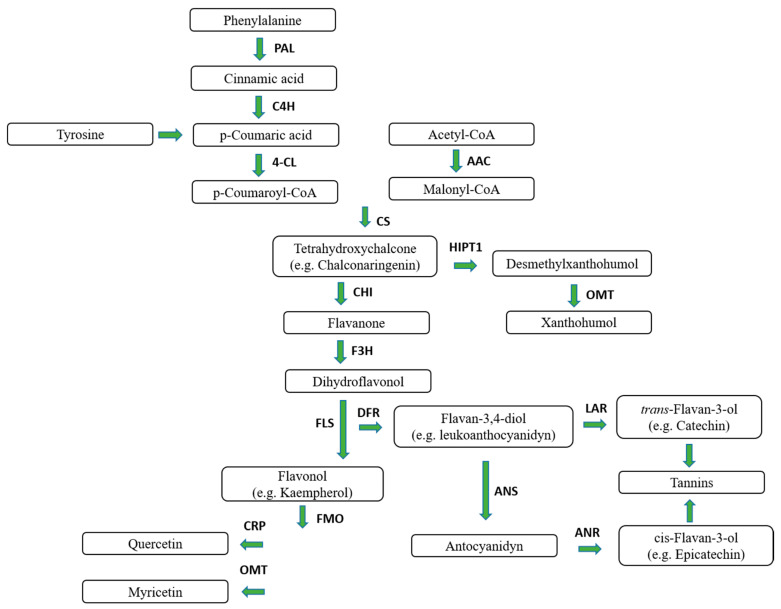
Biosynthesis pathways of several hop flavonoids. PAL: Phenylalanine ammonia-lyase; C4H cinnamate 4-hydroxylase, 4CL 4-coumarate-CoA ligase, AAC acetyl-CoA carboxylase, CS chalcone synthase, HIPT1 *Humulus lupulus* prenyltansferase-1,CHI chalcone isomerase, OMT *O*-methyltransferase, F3H flavanone 3-hydroxylase, FLS flavonol synthase, DFR dihydroflavonol 4-reductase, LAR leucoanthocyanidin reductase, ANS anthocyanidin synthase, ANR anthocyanidin reductase, FMO flavonoid 3-monooxygenase, CPR cytochrome P450 reductase, F3′5′H flavonoid 3′5′-hydroxylase.

Hops are rich in phenolic compounds, mostly flavonoids, which are secondary metabolites (Figure 1). Mass spectrometry analysis shows that the plant contains about 14.4% phenolic acids, flavonoids, proanthocyanidins, prenylated chalcones and flavanones as well as catechins [5]. In addition, malt accounts for 70–80% of all polyphenolic compounds found in beer [6].

### 1.1. Hop Prenylflavonoids

Flavonoids are phenolic derivatives with a flavan or chalcone flavonoid skeleton and have many beneficial effects on human health [7,8]. This is especially true of prenylflavonoids, which have so far been identified in 37 of plant genera, mainly in the families of *Leguminosae*, *Moraceae*, *Guttiferae*, *Umbelliferae*, *Rutaceae* and *Cannabaceae* [9].

These compounds differ in the position of the prenyl group, the number of attached isoprenoid units and modifications of the prenyl moiety, such as cyclization and hydroxylation [10].

**Figure 2:**
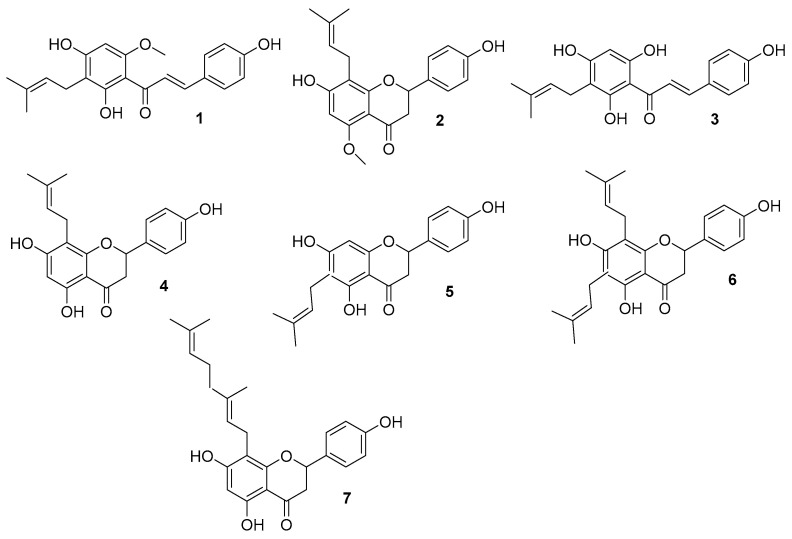
Structures of described flavonoids: xanthohumol XN (**1**), isoxanthohumol IX (**2**), desmethylxanthohumol DMX (**3**), 8-prenylnaringenin 8-PN (**4**), 6-prenylnaringenin 6-PN (**5**), 6,8-diprenylnaringenin 6,8-DPN (**6**), 8-geranylnaringenin 8-GN (**7**).

Prenylated flavonoids are accumulated in lupulin glands that cover bract of female hop cones. Their common precursor compound xanthohumol (XN) (**1**) (Figure 2), a prenylated chalcone, is the principal prenylflavonoid in the female flowers of the hop plant *Humulus lupulus* L. and provides 80–90% of its total flavonoid content [11].

XN (**1**) was extensively studied in the past and is considered the most active ingredient in hop and beer. It demonstrated chemopreventive activity [12], inhibiting initiation and development of carcinogenesis in human body [13,14] and therapeutic activity, by inducing cytotoxic effect [13] and apoptosis in the existing tumour cells [15,16,17]. In addition, it positively affects glucose and lipid metabolism [18,19,20], hepatic and intestinal metabolism and many others activities [21,22].

During beer production high temperature causes isomerization XN (**1**) toIXN (**2**) which become a major prenyl flavonoid present in beer; however, in the hydrophobic environment of plant cells, isomerization of XN (**1**) to IX (**2**) was not observed, hence hop’s prenyflavanones are represented by IXN (**1**), which is only about 0.01% of dry matter [23].

8-PN (**4**) has been identified in an amount of 0.002% of dry matter of hops [11], as well as 6-PN (**5**) which is present in slightly higher amounts (0.01%) [24]. Both compounds (**4**, **5**) are produced from DMX (**3**) in small quantities in a non-enzymatic manner during the drying, storage and extraction of hops.

### 1.2. Prenylflavonoids in Beer

**Figure 3:**
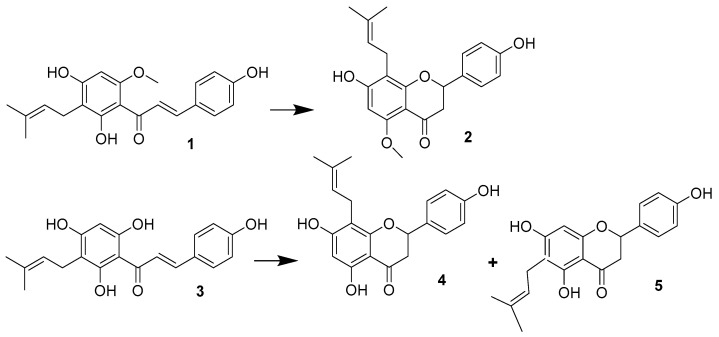
Paths of formation of IXN (**2**), 8-PN (**4**) and 6-PN (**5**) during wort boiling.

The use of high performance liquid chromatography and posteriori ultrasonic separation has shown that fermentation, boiling and the amount of hops used in beer production have a significant effect on the concentration of polyphenols in the final product [25]. During wort boiling in the brew kettle, the composition of the prenylflavonoids contained in it also changes. Only traces of the main hop of chalcone, XN (**1**) were found in the beer, and its final concentration does not exceed µg/mL [26]. During the brewing process 50 to 75% of this chalcone (**1**) isomerizes to IXN (**2**) (Figure 3), which is the main prenylflavonoid of beer [27]. Otherwise, the XN (**1**) precursor, DMX, (**3**) spontaneously is transformed into a mixture of (±) 6-PN(**5**) and the 1:1 racemate of (±) 8-PN(**4**) (Figure 3).

According to, the prenylflavonoid content in different beers (Table 1) can be significantly different: for XN (**1**) from 2 to 690 µg/L, for IX (**2**) from 40 to 3440 µg/L, for 8-PN (**4**) from 1 to 240 µg/L [11] and for 6-PN (**5**) from 7 to 200 µg/L [28]. The total content of hop flavonoids can increase to 5 g per 100 g of dry cones [29], while the total daily intake of hop prenylflavonoids can reach 0.14 mg, and their main source in the diet is beer [30].

Although it is XN (**1**) that is the major flavonoid found in hop (up to 1%), during wort boiling it undergoes thermal isomerisation to IXN (**2**), which becomes the major hop flavonoid in beer (up to 3.44 mg/L) [11,31] and therefore also in human diet.

## 2. Metabolism

The consumption of hop-derived products, especially beer and dietary supplements, is primarily responsible for human exposure to IX (**2**), 8-PN (4) and 6-PN (**5**) flavonoids, as well as to XN (**1**) chalcone. Pharmacokinetic studies showed that these flavonoids are rapidly conjugated with glucuronic acid [32].

### 2.1. Metabolism of IX *(**2**)*

XN (**1**) is converted to IX (**2**) via an acid-catalyzed reaction in the stomach. Nicolic et al. described 10 human liver microsomal metabolites of IXN (**2**) being the products of oxidation, isomerisation and demethylation, and one of them was 8-PN (**4**) [33]. IXN (**2**) demethylation occurs in the distal colon with an 80% efficiency using the intestinal microflora [34]. 8-PN (**4**) can also be derived from IX (**2**), by human liver CYP1A2 enzymes, as described by Guo et al. [35].

The above information indicates that the effect of 8-PN (**4**) on the human body does not depend directly on its dose in the diet. You should also take into account the amount of XN (**1**) (precursor of IX (**2**)) and IX (**2**) consumed, which is considered the main source of phytoestrogens in the diet. In addition, although the concentration of phytoestrogens in beer seems marginal, it can have a beneficial effect health effect and therefore need further pharmacological elucidation.

### 2.2. Metabolism and Bioavailability of Hop Estrogen

Flavonoids display several biological activities, but exhibit poor oral absorption and rapid metabolism. So far, little is known about the absorption, metabolism, and excretion of major hop phytoestrogens (8-PN (**4**) and/or 6-PN (**5**)) in humans. Only a few studies were conducted to clarify these problems, and low oral bioavailability and high inter-individual variability were found [36,37,38,39,40].

**Figure 4:**
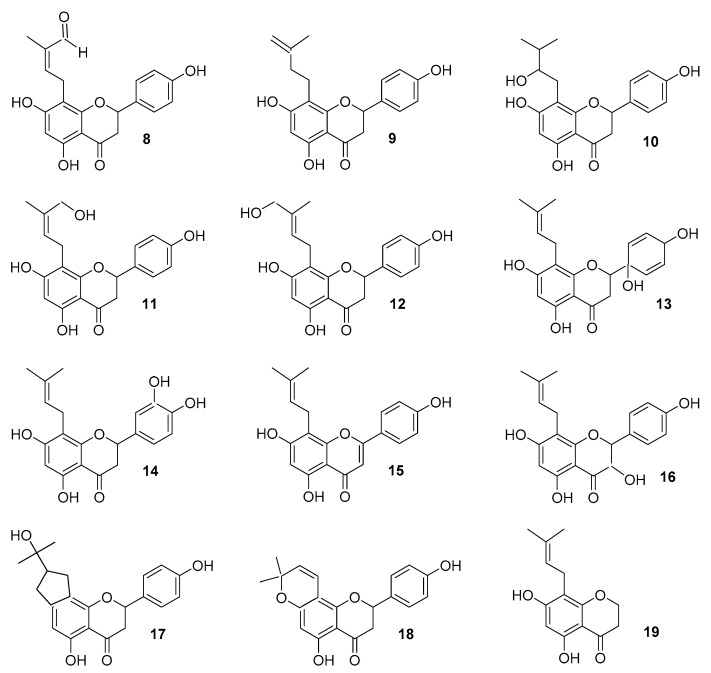
Human liver microsomes metabolites (**8**–**19**) of 8-PN (**4**) [40].

The half-life of racemic (2*R*,2*S*) 8-PN (**4**) from hops and from dietary supplements is over 20 h [41]. Nikolic et al. studied the metabolism of 8-PN (**4**) in vitro in human liver microsomes. They identified a total of 12 phase I metabolites, in which biotransformation occurred in the prenyl group and the flavanone skeleton, and two of them have estrogenic activity (Figure 4) [42]. In phase II, the glucuronide reaction dominates and mainly 8-PN (**4**) glucuronides are formed. Tests with purified human liver enzymes and microsomes have shown, inter alia, that both (2*R*)- and (2*S*)-8-PN-7-*O*-glucuronide formation dominated over 8-PN-4′-*O*-glucuronides, except for the UGT1A10 intestine, which produced more (2*S*)-8-PN-4″-*O*-glucuronide. These data mean that phenotypic differences in humans can affect the metabolism pathways of this estrogen [41].

The pharmacokinetic profile of 8-PN (**4**) has been described by Rad et al. based on observations of 24 postmenopausal women who received 750 mg of estrogen orally. The maximum plasma concentration (C_max_) of the compound was 110 nmol. The maximum plasma concentration of (**4**) was reached within 1–1.5 h, and the second peak occurred after 7–10 h as a result of enterohepatic circulation [43]. 8-PN (**4**) does not undergo significant conversion during phase I metabolism [44]. After absorption from the gastrointestinal tract, hepatic metabolism includes phase II dominating glucuronidation and sulfation reactions. In addition, large differences in maximum plasma concentrations were observed between different individuals, most likely due to polymorphism of the enzymes involved in metabolism [45]. A linear relationship between dose and maximum blood concentration was confirmed. In humans, metabolites of 8-PN (**4**) are mainly excreted in faeces and bile because no significant metabolites have been detected in urine [40].

Calvo-Castro et al. studied the bioavailability and safety of oral administration of 6-PN (**5**) and 8-PN (**4**) in 16 healthy young women and men, as well as their effect on peripheral blood mononuclear cells (PBMCs). Experiments have shown that 8-PN (**4**) is much more bioavailable than its isomer **5** in healthy people, but both compounds are similarly effective in increasing PBMC viability [45].

The metabolism and mode of action of all prenylated flavonoids is not yet fully understood—especially the biotransformation of those compounds that occur in hops in small amounts.

## 3. Phytoestrogens

Phytoestrogens are non-steroidal plant-derived substances that show remote structural and definite functional similarities to estradiol (**20**).

Estrogen hormones are primarily involved in regulating the functioning of the reproductive system and central nervous system. They also affect the maintenance of proper bone density, water retention, blood clotting and stimulate growth. The degree of estrogenic activity is generally determined by their affinity for receptors, the presence of transcription coactivators or promoter correctors [46]. There are two types of estrogen receptors (ER) in humans; ERα occurs mainly in the endometrium, ovarian stroma, bones and mammary gland, and ERβ occurs mainly in adipose tissue, endothelial cells, brain, kidneys and prostate [47,48]. The substrates usually have preferential affinity for one type of ER receptor [49], but the affinity of estradiol (**20**) for both types of receptors is roughly the same [50]. In addition to the ER receptor activating pathway, phytoestrogens may also act through non-genomic mechanisms, via interaction with cell surface receptors or by epigenetic mechanisms [51].

Beside the lignans, the isoflavones represent the main class of phytoestrogens. In contrast to less investigated other groups of phytoestrogens, e.g., coumestans and stilbenes, which in a lower amount are contained in food, isoflavones are well studied. The most important representatives of this class of phytoestrogens are genistein, daidzein and glycine, but the strongest phytoestrogen is 8-PN (**4**) present in hop and beer [52].

### 3.1. Hop Phytoestrogens and Their Activities

The estrogenic effect of hop has been recognized for decades, the first mention of the estrogenic properties of this plant dates back to 1953, by Koch and Heim [53]. Inspired by this report, Bednar and Zenisek (1961) were interested in the possibility of using these natural estrogen products for use in cosmetics. Since then, several cosmetic preparations containing hop have been patented [54]. Nastainczyk in 1972 reported the isolation of “hops proestrogen” (3′-prenylnaringenin chalcone), synonymous with DMX (**3**), which isomerizes into a mixture of “hops estrogen” [55]. It was not confirmed until 1988 when Hänsel and Schulz were provided for the first time the chemical structure of “hops proestrogen” (a mixture of 8-PN (**4**) and 6-PN (**5**)) [56].

**Figure 5:**
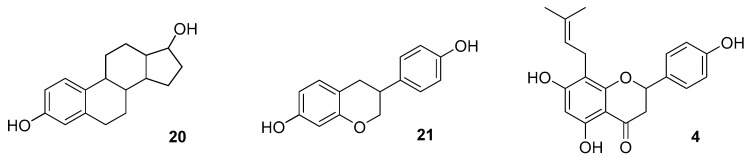
Comparison of chemical structures of estradiol (**20**), isoflavone (equol, (**21**)) and 8-PN (**4**) [63].

Milligan et al. using Ishikawa cells for screening, isolated and characterized 8-PN (**4**) as the major estrogenic substance in hops and one of the strongest known plant-derived estrogens [52,57]. In vitro studies have shown that 8-PN (**4**) mimics the effects of 17β-estradiol (**20**) (Figure 5), although phytoestrogen has many times lower (10–20,000 times) potency [58,59,60,61,62].

Schaefer et al., reported that 8-PN (**4**) has a higher affinity for ERα, where it is 70 times weaker than estradiol (**20**) for ERβ, reported as 20,000 times weaker than estradiol (**20**) [64]. For 2*S* and 2*R* enantiomers, no significant difference in ER binding strength was observed [65]. The prenyl group is probably responsible for the high strength and efficiency of binding to ERα because naringenin has no 8-prenyl side chain and is much weaker than 8-PN (**4**). Prenylation increases the hydrophobicity of phytoestrogen, which optimizes binding in the hydrophobic pocket of ERα [66].

The majority of known phytoestrogens have a higher affinity for the ERβ receptor [67], while 8-PN (**4**) binds mainly to ERα, with approximately 100 times higher affinity compared to isoflavone-genistein [68,69].

The concentration of 8-PN (**4**) in hormone sensitive tissues (e.g., endometrium, mammary gland) may be much higher due to active transport through ERα [70]. In addition, although the concentration of 8-PN (**4**) in raw hop extracts is at least 10 times lower than XN (**1**) [70], but in vivo more is formed from XN (**1**), as well as from IX (**2**), which is the major flavonoid in the diet.

In contrast to the most active 8-PN (**4**), other structurally related hop flavonoids, such as 6-PN (**5**), 6,8-DPN (**6**) and 8 GN (**7**), have poor estrogenicity. Their activity was less than 1% compared to 8-PN (**4**) [59].

Menopause was defined as the last menstrual period caused by permanent cessation of menstruation due to the end ovarian of follicular activity. During menopause estrogen production is declining, and leads to physiological symptoms such as irregular bleeding, hot flashes, loss of libido, night sweats, tachycardia, breast pain, lack of energy, dyspareunia, joint soreness, atrophic vaginitis, interrupted sleeping patterns, anxiety, mood swings, and dry skin [71]. Menopausal women are also more susceptible to osteopenia and osteoporosis [72] and atherosclerosis [73,74].

Sarcopenia, age-related muscle mass loss caused by decline in androgens, estrogens and progesterone affects more than 30% of individuals over 60 years of age [75]. Especially in case of postmenopausal women, who experience a greater decline in muscle strength than do men of similar age, this disorder has a great impact on physical function [76]. Although numerous studies focused on the effects of sex hormones on sarcopenia, still many molecular mechanisms in skeletal as well as smooth muscle cells remain to be investigated. Mukai at al. proved that intake of the 8-PN for 14 days in mixed diet accelerated recovery from muscle atrophy, and prevented reductions in Akt phosphorylation. These data clearly suggest that 8-PN might be a candidate to be used as a supplement to aid recovery from disuse muscle atrophy [77].

Menopause symptoms can be eliminated with the help of hormone therapy. A 2002 WHI (Women’s Health Initiative) study found that hormone replacement therapy (HRT), although preventing fractures, has life-threatening side effects, including an increased risk of cancer, stroke and atherosclerosis [78] and is currently not recommended. Various symptoms associated with menopause can be successfully resolved by replacing HRT with alternative agents such as botanicals dietary supplements e.g., soybean (*Glycine max*), red clover (*Trifolium pratense*), kudzu (*Pueraria lobata*), hops (*Humulus lupulus*), licorice species (*Glycyrrhiza species*), rhubarb species (*Rheum rhaponticum*), flaxseed (*Linum usitatissimum*), alfalfa (*Medicago sativa*) and *Epimedium* species [79].

Extracts from spent hops are rich in bioactive prenylated flavonoids that have a positive effect on health. XN (**1**), proestrogens (IXN (**2**), DMX (**3**)) and phytoestrogens (mainly 8-PN (**4**) and 6-PN (**5**)) are present in hops, but if we want to use hops extracts, it is necessary to know and understand the biological activity of the compounds contained from them and to develop standardized botanical products with higher efficiency, safety and chemopreventive properties.

In developed countries, postmenopausal osteoporosis is now a very serious problem that will get worse in the future. Osteoporosis is characterized by reduced bone mass and damaged bone structure. It occurs mainly in women and is associated with aging and hormone deficiency which increases bone resorption [80]. Numerous forecasts and aging of the population indicate that in the next decades there will be a significant increase in typical osteoporotic fractures [81].

Alternative osteoporosis treatment is still being sought, and interest in phytoestrogens has recently increased. Studies on the effects of the strongest phytoestrogen on osteoporosis are scarce and ambiguous. Some have shown that 8-PN (**4**) has osteoprotective effects on the tibia [64,82,83,84] while Hoffmann et al. did not confirm the protective effect of 8-PN (**4**) at a safe dose due to cancer in primary bone osteoporosis [85]. Keiler et al. provided evidence of the safety of using standardized hops extract (no proliferation in the endometrium) and showed a weak protective effect of the extract on bone loss after estradiol (**20**) depletion in ovariectomized rats [86].

In the rat model, it was observed that musculoskeletal symptoms are caused by a slight increase in body temperature combined with a smaller thermo-neutral zone [87]. These processes are controlled by the area of the anterior hypothalamus, which reacts to sex hormones. The effect of phytoestrogen (8-PN (**4**)) was studied in the same model—by measuring the tail skin temperature (TST) of rats whose ovaries were removed compared to the control group. Normal TST was restored after subcutaneous or oral administration of low doses of 8-PN (**4**), suggesting that temperature irregularities during menopause may be due to changes in the level of sex hormones in the regulatory system. The obtained results suggest that the use of 8-PN may reduce hot flashes in postmenopausal women [88]. Hops phytoestrogens may also affect other ailments in menopausal women.

Based on preliminary data and subsequent in vivo experiments, preclinical trials were initiated. Several of them assess the effectiveness of hops extracts during menopause.

Heyerick et al. tested standardized hop extract at a dose of 100 µg/day or 250 µg/day for 12 weeks for its ability to relieve menopause discomfort in 67 menopausal women. At a dose of 100 µg/day there was a significant decrease in Kupperman Index (11-element questionnaire used to assess the effectiveness of therapy) compared to placebo after 6 weeks, which shows the potential of hop substances in ameliorating menopause symptoms, but no effect was observed at higher doses [89].

Another prospective, double-blind, placebo-controlled study showed that placebo was also effective for 8 weeks after active treatment with the extract for 8 weeks (10 μg 8-PN/day) [90]. Significant efficacy of hop and no adverse effects were observed in 120 patients receiving 500 mg dried hop tablets containing 100 μg phytoestrogens (the exact content of 8-PN (**4**) in the tablets was not specified) for 90 days [91].

Although some tests are positive, the results so far may not be reliable, given the small sample size and the large number of variables included in the statistical analysis [92,93].

Further clinical studies are needed to determine the safety and efficacy of hops extracts in relieving menopause. Side effects after long-term use of hop phytoestrogens can be serious and occur early.

Keiler et al. reported that low doses of 8-PN (**4**) increase libido women [94], but in vivo studies showed that 8-PN (**4**) and naringenin significantly affect the maturation of swine oocytes by reducing cumulative expansion. In addition, both flavanones reduce the percentage of meiotic spindle formation, oocyte cleavage and blastocyst formation. The flavanone induced effects were observed at concentrations that can be found in human plasma after taking the supplement and which resemble physiological levels of estrogen equivalence in follicular fluids. Improper maturation of oocytes can cause infertility, so it is recommended to take precautions and avoid excessive consumption of dietary supplements containing phytoestrogens, as they may have a negative effect on reproduction [95].

Although excessive consumption of phytoestrogen-containing dietary supplements is not recommended, women may take 8-PN (**4**), which is present in breast enhancement supplements. However, this effect has not been confirmed yet in clinical trials [96].

#### 3.1.1. Protective Function on the Cardiovascular System

8-PN (**4**), like all phytoestrogens in food, protects people against the risk of cardiovascular disease, inhibits platelet aggregation and adhesion in independently of ER’s [97]. Antiatherosclerotic effect of 8-PN (**4**) was evaluated in a study of 157 postmenopausal women who were given the herbal preparation for 12 months. The preparation contained a mixture of plants rich in isoflavonoids, powdered hop cones and powdered garlic, and its consumption had a positive effect on the thickness of the intima media of the common carotid arteries and inhibited the growth of atherosclerotic plaques [98].

#### 3.1.2. Neuroprotection

Hops have neuroactive properties as a sedative and hypnotic; however, not all compounds responsible for this activity have been fully understood in terms of their pharmacological properties. XN (**1**), IX (**2**) and 8-PN (**4**) have been shown to positively modulate GABA-induced responses at native and recombinant GABAA αβγ receptors at low micromolar concentrations [99].

It was observed that 8-PN (**4**) administration prevented gastrocnemius muscle weight loss in a mouse denervation model. In addition, the activity of this molecule was due to the presence of a prenyl group [100].

Neurodegenerative diseases are an increasing burden on aging societies, which is why new effective drugs are constantly being sought. The greatest possibility of endogenous brain repair and functional regeneration is neurogenesis, which in adults is partly under the control of sex hormones such as estradiol (**20**). Increased neurogenesis in animals and improvement of cognitive function was observed in animals after administration of estradiol (**20**). Phytoestrogens, including 8-PN (**4**) and 6-PN (**5**) were tested which showed that estrogen activity does not correlate directly with the activity of induction of differentiation in neuronal precursor cells [101].

#### 3.1.3. Anticancerogenic Activities

In recent years, attention has been paid to prenylflavonoids and their metabolites, which have anti-cancer potential. Due to their antioxidant activity [102,103,104], they protect by modulating biotransformation of carcinogens, and they can also act antiproliferatively, inhibit angiogenesis, effect on the cell cycle and induce apoptosis in cancer cells.

Cancer is one of the main causes of death in industrialized countries [105] and in women one of the most commonly diagnosed cancers is breast cancer. Exposure to estrogen has long been considered one of the risk factors associated with the development of the disease, especially in postmenopausal women. Menopausal women often use hormone replacement therapy. However, HRT may increase the risk of disease, which is why some women choose supplements containing phytoestrogens as a natural alternative.

However, their influence on the risk of estrogen-dependent cancer development is not unequivocal. Phytoestrogens may be implicated in the etiology of breast cancer, and are being evaluated as potential cancer chemopreventive or promoting agents [106,107].

Phytoestrogens may serve as chemopreventive agents, while promoting the growth of cancer cells using the estrogen receptor. In addition, they can exert estrogenic effects through receptor dependent and/or receptor independent mechanisms. ERα activation is associated with proliferative responses in the mammary gland and uterus. These findings suggest that consumption of phytoestrogens may not be appropriate for patients at increased risk of hormone-dependent cancers or survivors [108]. Although phytoestrogens administered during menopause can stimulate endometrial and mammary gland tissue proliferation, it is also known that Japanese women who consume a lot of phytoestrogens are less likely to experience breast cancer [109].

Molecular docking study showed that almost all popular herbal supplements contain phytochemicals that can bind to the human estrogen receptor. For example, estrogens can be effective stimulators of the growth of estrogen receptor positive tumors and also pose a hazard to patients who have ER-positive tumors and who are being treated with antiestrogens. The strongest docking plant ligands were phenolic compounds and the weakest docking ligands were triterpenoids [110].

Hop cones extracts used in the production of beer are present in dietary supplements to treat postmenopausal symptoms and other ailments. Researchers are investigating whether the plant extracts can also help fight breast cancer because in recent years, numerous in vitro (Table 2) have shown the antitumor potential of hops flavonoids.

Research to date has shown that 8-PN (**4**) against estrogen-dependent breast cancer cell lines (T-47D, MCF-7) is less active than 6-PN (**5**) [111], sometimes induces apoptosis [112], and even showed little up-regulation of metabolism in MCF-7 cells [113,114]. Wang et al. showed that 6-PN (**5**) preferentially induces CYP1A1 P450 mRNA expression, and increases P4501A1/1B1 activity, which means that 6-PN (**5**) preferentially enhances the non-toxic 2-hydroxylation pathway of estrogen in the MCF-10A and MCF-7 lines [114].

Probably 6-PN (**5**) has greater potential for use in estrogen-dependent cancers, but little is known so far about the pharmacological properties of this phytoestrogen.

Dietz et al. examined tissue-specific activity for XN (**1**) and 8-PN (**4**) and showed that XN (**1**) induces the detoxification enzyme quinone oxidoreductase NAD(P)H (NQO1) in hepatic tissue, so it exhibits cytoprotective activity, whereas 8-PN (**4**) is responsible for the reduction of NQO1 in the mammary gland [70]. Properly developed hops extract, i.e., containing a reduced level of estrogen 8-PN (**4**) and a higher concentration of chemopreventive XN (**1**), can be intended for women in premenopausal period. The extract intended for postmenopausal women would be slightly changed [115].

Tan et al. (2014) tested the interaction between XN (**1**), IX (**2**), 8-PN (**4**), 6-PN (**5**), 6,8-DPN (**6**) and a resistance protein to breast cancer (BCRP/ABCG2). This protein is an important transporter of xenobiotic bioavailability and multiple drug resistance (MDR) [125].

Studies have shown that **1**, **2**, **4**, and 8-PN sulfate are potent ABCG2 inhibitors, and therefore are involved in food/herbal interactions through ABCG2 and MDR. The action of test compounds by ABCG2 may represent one of the mechanisms regulating the bioavailability of prenylflavonoids [125]. On the other hand, ABCG2 is involved in MDR in cancer chemotherapy [126] therefore, caution should be exercised when co-administering prenylflavonoid-rich foods/dietary supplements with ABCG2 substrates. Increased absorption and distribution of flavonoids due to the importance of ABCG2 may reverse MDR activity [125].

#### 3.1.4. Anti-Inflammatory Activity

In the last few years, adverse effects of currently used drugs (coxibs) [127] and estrogens on the cardiovascular system have been reported [128].

In search of new suitable compounds for use in chronic therapies, attention has been paid to natural flavonoids [129].

The anti-inflammatory mechanisms of bioactive beer compounds are mainly caused by inhibition of inducible iNOS (nitric oxide synthase) and inhibition of the activity of COX-1 (cyclooxygenase 1). Probably the main anti-inflammatory effect, mediated by inhibition of iNOS induction, is caused by XN (**1**). In addition, XN (**1**) and humulone exert an anti-inflammatory effect by inhibiting the endogenous synthesis of prostaglandin E2 by COX-2 induced by TNFα [21,59].

Phytoestrogens with structural [130] and pharmacodynamic properties act similary to estrogens [131] and are not good anti-inflammatory compounds. The anti-inflammatory and anti-angiogenic properties of XN (**1**), IX (**2**) and 8-PN (**4**) were tested in vivo using them in the rat skin wound healing test. Both histopathological and immunochemical tests confirmed the anti-inflammatory and anti-angiogenic effects of **1** and **2**, while **4** acted as a strong pro-inflammatory and angiogenic factor [132].

However, Paoletti et al. in the in vitro model showed that slight modification of the structure (e.g., 8-prenylapigenin) may be a rational design of new anti-inflammatory and blood vessel protecting compounds [133].

#### 3.1.5. Anti-Diabetic Activity

In recent years, XN (**1**), IX (**2**) and 8-PN (**4**) have been extensively tested for their anti-diabetic properties [96,134,135]. Costa et al. tested XN (**1**) and 8-PN (**4**) in a mouse model and found that both compounds have the potential for therapeutic intervention in diabetics type 2 [134].

#### 3.1.6. Antimicrobial Activity

The antimicrobial potential includes antibacterial, antiviral, antifungal, and antiparasitic activities. Hops contain many compounds with antibacterial activity. β and α acids have the highest activity, but only in undissociated forms [136]. The potential antimicrobial activity of the hop compounds is mainly dependent on the hydrophobic parts of these molecules (prenyl- and acyl-chains) and results from an interaction between these hydrophobic parts and the bacterial cell wall [137] or reduces bacterial adhesion on abiotic surfaces, inhibiting biofilm formation [138].

6-PN (**5**) has shown very interesting antibacterial activity [21,139], although its mode of action has not yet been explained.

Some hop flavanones have antimicrobial properties, including antifungal and antibacterial activity for 6-PN (**5**) and antiviral activity for IXN (**2**), while chalcone XN (**1**) is the most active. Gerhauser et al. [21] showed that among of tested hops flavonones (naringenin, IXN (**2**), 6-PN (**5**) and 8-PN (**4**)) the most active compound towards *Staphylococcus aureus*, with minimal inhibitor concentration was 6-PN (**5**) (6.25 g/mL). Bartmańska et al. [140] proved that 8-PN (**4**) against *S. aureus* (MSSA) was more active (MIC=12.5 µg/mL) than IXN (**2**), in this studies 6-PN (**5**) was not tested. There are also evidences that phytoestrogens from hops have antifungal activity against *Candida albicans*, *Fusarium oxysporum*, *Mucor rouxianum*, *Trichophyton mentagrophytes*, *Trichophyton rubrum* [139] as well as they are antiviral agents, and can inhibits growth of *Plasmodium falciparum* [141].

These prenylflavonoids are excellent candidates for further research to fully understand pharmacokinetic and pharmacodynamic properties. The next step may be to consider using them for food preservation as well as natural pharmaceutical products.

The Fusarium toxin: zearalenone (ZEN) and its metabolite α-zearalenol (α-ZEL) often contaminate cereal products, including beer, and disrupt the hormonal levels of organisms. However, hops and beer are a source of endocrine active compounds, including XN (**1**), DMX (**3**), IX (**2**), which can be converted into a strong 8-PN (**4**) phytoestrogen. In vitro studies have shown that XN (**1**) acts as a potent antagonist of mycotoxin-induced estrogenicity, significantly suppressing the AlP-inducing effect of fungal metabolites, and 8-PN (**4**) has weaker activity [142].

### 3.2. Application

The use of botanical natural health products and dietary supplements are still increasing. Products containing hops (hop phytoestrogens) are used, among others, for menopause support, hormone replacement therapy, breast enhancement.

Among the many products containing hops available on the Polish market are: Ligunin termostop, Doppelherz aktiv Aktiv-Meno, Doppelherz aktiv Aktiv-Meno Forte, Climea forte, Menopauzin, Menopauzin Forte, Duo-Fem, Solgar, but only some of them deserve attention—it is necessary to provide information on the amount of active substance. A good example is the Ligunin thermostat—two capsules (daily dose) contain 170 mg of hops extract, including 70 µg 8-PN (**4**).

## 4. Conclusions

The prevention and control of chronic diseases such as cardiovascular disease, Alzheimer’s disease and many cancers will be one of the most important medical challenges in the years to come. Scientific and clinical discoveries regarding the biological activity of hops indicate the possibility of wider use of this plant in both medicine and nutrition. New uses as food for health are just as possible as the processing of individual products in pure form for use as dietary supplements and medicines. Phytonutrient supplements are a promising complementary therapy in the treatment of chronic diseases. Among them, *Humulus lupulus* L. has paid special attention worldwide because it contains many phytochemicals.

Our review provides insight into the unusual hop phytoestrogens that are prenylated flavonoids. In addition to their role in the plant, they also show numerous health benefits associated with their wide spectrum of biological activities, and are therefore intensively studied.

The strongest known phytoestrogen is 8-PN (**4**), a compound produced by lupulin glands (*Humulus lupulus* L.) and present in small amounts (less than 20 µg/L) in beer. This molecule has ER-dependent activity in mammalian cells at a concentration of about 1.0 μM. Other hop prenylflavonoids with low estrogenic activity are 6-PN (**5**), 6,8-DPN (**6**) and 8-PG (**7**).

These compounds have a beneficial effect on health, and their most important properties are estrogen and anticancer. 8-PN (**4**) shows positive results in several in vitro tests that assess potential beneficial effects. The effects on type 2 diabetes, as well as other effects, require complementary studies. Phytoestrogen 6-PN (**5**) has anticancer activity against cancer cell lines with a positive estrogen receptor, and it is a very interesting compound due to its antimicrobial properties, too.

It is necessary to strictly determine the bioavailability of phytoestrogens in vivo and to carry out toxicological studies on all their transformation products, especially in humans.

There is a great need for agents for the treatment of HRT. Hops phytoestrogens, especially 8-PN (**4**), have strong estrogen-like effects, therefore they seem to be a good alternative to HRT, but it is difficult to assess long-term effects for the patient.

Each case should be considered individually, adjusting the woman’s age, duration of therapy, dose, route of administration and the exact type and combination of hormones. The chances of achieving treatment goals in relation to the risk of an adverse event should also be assessed.

Hop components are responsible for various effects, including those related to hormonal, metabolic, inflammatory, and epigenetic pathways. To be able to use hop extracts as supplements, it is necessary to fully understand the biological activity of all compounds contained in them. Preparations with extracts of hops have the status of supplements, contain not only estrogens, but also proestrogens, which makes it difficult to precisely determine the doses used, and therefore can lead to uncontrolled self-healing. To be able to use hops extracts as supplements, it is necessary to fully understand the biological activity of all compounds contained in them and to develop standardized botanical products with higher efficiency, safety, and expected properties.

Such hops extracts should contain corrected doses of the desired bioactive phytochemicals, including chemopreventive XN (**1**), and should be free of undesirable phytochemicals that can expose people to side effects. However, for medicinal purposes, the use of pure compounds seems safer.

Clinical trials aim to assess their effectiveness in alleviating menopause and safety. A full explanation of the scope of action of phytoestrogens on the human body will contribute to the most beneficial the use of these compounds in the treatment of postmenopausal symptoms with minimized side effects will facilitate the choice of dose and duration therapy.

Researchers are always looking for new molecules that maximize the desired effects of therapy, minimizing or excluding risk. To this end, already known basic structures of compounds can be modified. In the future, treatment may be based on individual gene mapping and careful assessment of the chance of achieving treatment goals in relation to the risk of an adverse event.

## Figures and Tables

**Table 1:** Prenylflavonoids contents in different type of beers.

Contents (μg/L) and Type of Beer		XN (1)	IX (2)	8-PN (4)	Minor Prenylflavonoids (Including 6-PN (5) and 6-/8-GN (7))	6-PN (5)	References
**European Beers**	Stout	340	2100	69	2680		[11,28]
	Lager	2	40	1	43		
	Pilsner	28	570	21	680		
		12	1060	8	1100		
**US Beers**	Lager/pilsner	34	500	13	590		
		9	680	14	750		
		14	400	17	460		
	Porter	690	1330	240	2900		
	Wheat	5	300	8	330		
	Strong ale	240	3440	110	4000		
	India pale ale	160	800	39	1160		
**Other**	Alcohol-free	3	110	3	120		
		3	100	3		7	
	Ale	100	2100	44	11 (6-GN)	200	
	Dark	300	1200	92	27 (6-GN)	200	
	Regular	14	400	10	46 (GN)	26	

**Table 2:** In vitro activity of phytoestrogens and their precursors as potential anticancer agents.

Biological Activity	Cell Line/Substrate	Main Active Components	Reference
Antiproliferative Activity	MCF-7, HT-29, A2780	IX	[13]
	PC-3, DU 145	DMX, IX, 6-PN, 8-PN	[14]
	T-47D A2780cis	6-PN, IX	[111]
	SK-MEL-28, BLM	6-PN, 8-PN	[116]
	PC-3, UO.31	6-PN, 8-PN	[117]
Induced Caspase-Independent Form of Cell Death	PC-3	IX, 6-PN	[118]
	DU 145	IX, 6-PN, 8-PN	
Induction of Quinone Reductase (QR) Activity	Mouse hepatoma Hepa 1c1c7 cells	6-PN, 8-PN	[119]
		IX	[120]
Antioxidant and Antiperoxidant	Isolated human LDL	DMX	[102]
Inhibition of Metabolic Activation of Procarcinogens	Rat liver microsomes CYP1A1, CYP1B1, CYP1A2, CYP3A4,CYP2E1	DMX	[121]
	P450 1A1 and 1B1	6-PN	[114]
	CYP1A2	IX, 8-PN	[122]
	CYP1A2	IX, 8-PN	[119]
	CYP1A	IX, 6PN, 8-PN	[120]
	MCF-7, CYP1A1	6-PN	[114]
Inhibition of Nitric Oxide Synthase (iNos)	Mouse macrophage cells	IX	[120]
Inhibition of Angiogenesis	Human placental vessels	IX	[123]
Induction of Apoptosis in Tumor Cells	MCF-7	8-PN	[112]
	HCT-116		[124]
Inhibition of Cyclooxygenase Enzymes: COX1	Sheep seminal vesicle microsomes	8-PN	[120]

MCF7, T-47D—human breast cancer cells lines; HT-29, HCT-116—human colon cancer cells lines; A2780 human ovarian cancer cells line; A2780cis—human cisplatin-resistant ovarian cancer cell line; PC-3, DU 145—human prostate cancer cells lines; UO-31—renal carcinoma cells, SK-MEL-28, BLM—human metastatic melanoma cells lines; LDL—low-density lipoprotein; CYP—cytochrome P450
